# Prediction and Feature Importance Analysis for Severity of COVID-19 in South Korea Using Artificial Intelligence: Model Development and Validation

**DOI:** 10.2196/27060

**Published:** 2021-04-19

**Authors:** Heewon Chung, Hoon Ko, Wu Seong Kang, Kyung Won Kim, Hooseok Lee, Chul Park, Hyun-Ok Song, Tae-Young Choi, Jae Ho Seo, Jinseok Lee

**Affiliations:** 1 Department of Artificial Intelligence The Catholic University of Korea Bucheon Republic of Korea; 2 Department of Trauma Surgery Jeju Regional Trauma Center Cheju Halla General Hospital Jeju Republic of Korea; 3 Radiology and Research Institute of Radiology Asan Medical Center University of Ulsan College of Medicine Seoul Republic of Korea; 4 Department of Internal Medicine Wonkwang University School of Medicine Iksan Republic of Korea; 5 Department of Infection Biology Wonkwang University School of Medicine Iksan Republic of Korea; 6 Department of Pathology Wonkwang University School of Medicine Iksan Republic of Korea; 7 Department of Biochemistry Wonkwang University School of Medicine Iksan Republic of Korea

**Keywords:** COVID-19, artificial intelligence, blood samples, mortality prediction

## Abstract

**Background:**

The number of deaths from COVID-19 continues to surge worldwide. In particular, if a patient’s condition is sufficiently severe to require invasive ventilation, it is more likely to lead to death than to recovery.

**Objective:**

The goal of our study was to analyze the factors related to COVID-19 severity in patients and to develop an artificial intelligence (AI) model to predict the severity of COVID-19 at an early stage.

**Methods:**

We developed an AI model that predicts severity based on data from 5601 COVID-19 patients from all national and regional hospitals across South Korea as of April 2020. The clinical severity of COVID-19 was divided into two categories: low and high severity. The condition of patients in the low-severity group corresponded to no limit of activity, oxygen support with nasal prong or facial mask, and noninvasive ventilation. The condition of patients in the high-severity group corresponded to invasive ventilation, multi-organ failure with extracorporeal membrane oxygenation required, and death. For the AI model input, we used 37 variables from the medical records, including basic patient information, a physical index, initial examination findings, clinical findings, comorbid diseases, and general blood test results at an early stage. Feature importance analysis was performed with AdaBoost, random forest, and eXtreme Gradient Boosting (XGBoost); the AI model for predicting COVID-19 severity among patients was developed with a 5-layer deep neural network (DNN) with the 20 most important features, which were selected based on ranked feature importance analysis of 37 features from the comprehensive data set. The selection procedure was performed using sensitivity, specificity, accuracy, balanced accuracy, and area under the curve (AUC).

**Results:**

We found that age was the most important factor for predicting disease severity, followed by lymphocyte level, platelet count, and shortness of breath or dyspnea. Our proposed 5-layer DNN with the 20 most important features provided high sensitivity (90.2%), specificity (90.4%), accuracy (90.4%), balanced accuracy (90.3%), and AUC (0.96).

**Conclusions:**

Our proposed AI model with the selected features was able to predict the severity of COVID-19 accurately. We also made a web application so that anyone can access the model. We believe that sharing the AI model with the public will be helpful in validating and improving its performance.

## Introduction

The COVID-19 pandemic has had a major impact on health care systems globally. Since early 2020, COVID-19 has spread rapidly around the world, exceeding 100 million cases and 2 million deaths [1]. In the COVID-19 pandemic situation, the most important issue in the management of COVID-19 patients is to triage patients at high risk of mortality and provide tailored treatment, so that medical costs and mortality rates can be reduced.

Several models have been proposed to predict the severity or mortality of COVID-19 patients using artificial intelligence (AI) techniques. The majority of them have been developed based on limited information or variables, such as medical images [2-7], blood and/or urine information [8,9], clinical characteristics [10-12], individual-level epidemiological data sets [13], and electronic health records (ie, demographics, laboratory results, medical history, and vital signs) during hospitalization [14]. However, most of them were developed based on relatively small samples from limited data sources, which makes their generalization problematic. More specifically, the numbers of patients used for training of some models were 375 [15], 443 [16], 548 [17], and 663 [18].

To overcome the generalization issue, we aimed to develop an AI prediction model based on confirmed nationwide patient data obtained from the South Korean government, which included 5601 patients from more than 100 hospitals. In this model, we used comprehensive data sets composed of 37 factors, including basic demographic information, vital signs, physical examination results, clinical symptoms and severity, comorbid diseases, and general blood test results. To the best of our knowledge, this is the first attempt to develop an AI model to predict the severity of COVID-19 based on a nationwide cohort and comprehensive data set in South Korea.

## Methods

### Data Sets

This study was approved by the Korea Disease Control and Prevention Agency (KDCA) in South Korea. Informed consent was waived. The KDCA has been managing comprehensive data from COVID-19–confirmed patients in Korea obtained from approximately 100 hospitals. The KDCA discloses this data to few selected researchers during a specific study period. Thus, we investigated this data between September 15 and October 5, 2020, under the approval of the KDCA.

Table 1 describes the KDCA data set. The basic patient information includes the patient’s ID, age, gender, outcome, quarantine period, pregnancy status, and pregnancy week. The physical index includes body mass index. The initial examination findings include systolic and diastolic blood pressure, heart rate average, and body temperature at the hospital admission stage. The clinical findings include the status of fever, cough, sputum production, sore throat, rhinorrhea, myalgia, malaise, dyspnea, headache, confusion, nausea, and diarrhea. The current or previous comorbid diseases include diabetes mellitus, hypertension, heart failure, chronic heart disease, chronic obstructive pulmonary disease, chronic kidney disease, cancer, chronic liver disease, rheumatism or autoimmune disease, and dementia. The clinical severity has two categories: low and high severity. The conditions of patients in the low-severity group correspond to no limit of activity, oxygen support with nasal prong or facial mask, and noninvasive ventilation. The conditions of patients in the high-severity group correspond to invasive ventilation, multi-organ failure with extracorporeal membrane oxygenation required, and death. The general blood test results include levels of hemoglobin, hematocrit, lymphocytes, platelets, and white blood cells.

Out of 5628 COVID-19 patient records, the clinical severity information was missing in 27 patient records, so we excluded them from our study. Thus, we used 5601 patient data records to develop the AI prediction model for clinical severity. For each patient data record, we used 37 variables as model inputs; these variables are summarized in Table 1 without ID, outcome, quarantine period, and clinical severity. As the model output, we used clinical severity, which is a binary component composed of low and high severity.

Table 2 summarizes the clinical features from the high-severity group (271/5601, 4.8%) and the low-severity group (5330/5601, 95.2%). Notably, in the high-severity group, 241 out of 271 patients were deceased (88.9%), while no patients died in the low-severity group.

**Table 1:** Description of COVID-19 patient data.

Item category and data			Type		Description
**Basic patient information**					
	ID	Number		Anonymous	
	Age (years)	9 categories		0-9 (0), 10-19 (1), 20-29 (2), 30-39 (3), 40-49 (4), 50-59 (5), 60-69 (6), 70-79 (7), ≥80 (8)	
	Gender	2 categories		Male (0), female (1)	
	Outcome	2 categories		Survived (0), deceased (1)	
	Quarantine period	Continuous		Days (0 if confirmed after death)	
	Pregnancy	2 categories		No (0), yes (1)	
	Pregnancy week	Number		Weeks (0 if not pregnant)	
Physical index: BMI (kg/m^2^)			5 categories		<18.5 (0), 18.5-22.9 (1), 23.0-24.9 (2), 25.0-29.9 (3), ≥30 (4)
**Initial examination findings**					
	Systolic blood pressure	5 categories		<120 (0), 120-129 (1), 130-139 (2), 140-159 (3), ≥160 (4)	
	Diastolic blood pressure	4 categories		<80 (0), 80-89 (1), 90-99 (2), ≥100 (3)	
	Heart rate	Number		Heart rate	
	Temperature	Number		Temperature	
**Clinical findings**					
	Fever	2 categories		No (0), yes if higher than 37.5 °C (1)	
	Cough	2 categories		No (0), yes (1)	
	Sputum production	2 categories		No (0), yes (1)	
	Sore throat	2 categories		No (0), yes (1)	
	Runny nose or rhinorrhea	2 categories		No (0), yes (1)	
	Muscle aches or myalgia	2 categories		No (0), yes (1)	
	Fatigue or malaise	2 categories		No (0), yes (1)	
	Shortness of breath or dyspnea	2 categories		No (0), yes (1)	
	Headache	2 categories		No (0), yes (1)	
	Altered consciousness or confusion	2 categories		No (0), yes (1)	
	Vomiting or nausea	2 categories		No (0), yes (1)	
	Diarrhea	2 categories		No (0), yes (1)	
**Current or previous comorbid diseases**					
	Diabetes mellitus	2 categories		No (0), yes (1)	
	Hypertension	2 categories		No (0), yes (1)	
	Heart failure	2 categories		No (0), yes (1)	
	Chronic cardiac disease	2 categories		No (0), yes (1)	
	Asthma	2 categories		No (0), yes (1)	
	Chronic obstructive pulmonary disease	2 categories		No (0), yes (1)	
	Chronic kidney disease	2 categories		No (0), yes (1)	
	Cancer	2 categories		No (0), yes (1)	
	Chronic liver disease	2 categories		No (0), yes (1)	
	Rheumatism or autoimmune diseases	2 categories		No (0), yes (1)	
	Dementia	2 categories		No (0), yes (1)	
Clinical severity			2 categories		Low severity, including no limit of activity, oxygen support required with nasal prong or facial mask, and noninvasive ventilation (0); high severity, including invasive ventilation, multi-organ failure, extracorporeal membrane oxygenation, and death (1)
**General blood test results**					
	Hemoglobin	Number		g/dL	
	Hematocrit	Number		%	
	Lymphocytes	Number		%	
	Platelets	Number		10^9^/L	
	White blood cells	Number		10^9^/L	

**Table 2:** Statistical summary of clinical features from the low-severity group and high-severity group (N=5601).

Participant data				Low-severity group (n=5330)		High-severity group (n=271)		*P* value
**Basic patient information**								
	Age category^a^, mean (SD)			4.26 (1.92)		7.05 (1.08)		<.001
	**Gender, n (%)**							<.001
		Male	2166 (40.6)		144 (53.1)			
		Female	3164 (59.4)		127 (46.9)			
	Pregnancy status (yes), n (%)			19 (0.4)		0 (0)		.33
	Pregnancy week, mean (SD)			16.50 (10.01)		N/A^b^		N/A
Physical index: BMI category^c^, mean (SD)				1.79 (1.02)		1.84 (1.13)		.54
**Initial examination findings, mean (SD)**								
	Systolic blood pressure category^d^			1.75 (1.31)		1.98 (1.46)		.008
	Diastolic blood pressure category^e^			1.00 (0.97)		0.90 (1.00)		.11
	Heart rate (beats per minute)			85.66 (14.79)		89.05 (19.64)		<.001
	Temperature (°C)			36.94 (0.54)		37.11 (0.80)		<.001
**Clinical findings (low-severity group n=5326), n (%)**								
	Fever			1197 (22.5)		105 (38.7)		<.001
	Cough			2239 (42.0)		92 (33.9)		.008
	Sputum production			1532 (28.8)		79 (29.2)		.89
	Sore throat			858 (16.1)		14 (5.2)		<.001
	Runny nose or rhinorrhea			609 (11.4)		8 (3.0)		<.001
	Muscle aches or myalgia			894 (16.8)		26 (9.6)		.002
	Fatigue or malaise			215 (4.0)		18 (6.6)		.04
	Shortness of breath or dyspnea			531 (10.0)		134 (49.4)		<.001
	Headache			946 (17.8)		17 (6.3)		<.001
	Altered consciousness or confusion			9 (0.2)		26 (9.6)		<.001
	Vomiting or nausea			226 (4.2)		18 (6.6)		.06
	Diarrhea			496 (9.3)		20 (7.4)		.28
**Current or previous comorbid diseases, n (%)**								
	Diabetes mellitus			582/5327 (10.9)		106 (39.1)		<.001
	Hypertension			1034/5327 (19.4)		164 (60.5)		<.001
	Heart failure			39/5327 (0.7)		20 (7.4)		<.001
	Chronic cardiac disease			150/5311 (2.8)		29 (10.7)		<.001
	Asthma			115/5327 (2.2)		13 (4.8)		.005
	Chronic obstructive pulmonary disease			31/5327 (0.6)		9 (3.3)		<.001
	Chronic kidney disease			37/5327 (0.7)		18 (6.6)		<.001
	Cancer			123/5326 (2.3)		22 (8.1)		<.001
	Chronic liver disease			76/5004 (1.5)		7 (2.6)		.17
	Rheumatism or autoimmune diseases			35/4998 (0.7)		3 (1.1)		.44
	Dementia			148/5001 (3.0)		76 (28.0)		<.001
**General blood test results, mean (SD)**								
	Hemoglobin (g/dL)			13.37 (1.69)		11.89 (2.23)		<.001
	Hematocrit (%)			39.51 (4.72)		35.28 (6.56)		<.001
	Lymphocytes (%)			30.08 (11.12)		15.08 (10.69)		<.001
	Platelets (10^9^/L)			239.96 (81.57)		188.51 (87.38)		<.001
	White blood cells (10^9^/L)			6.00 (2.55)		7.99 (5.10)		<.001

^a^Age categories were as follows (years): 0-9 (0), 10-19 (1), 20-29 (2), 30-39 (3), 40-49 (4), 50-59 (5), 60-69 (6), 70-79 (7), ≥80 (8).

^b^N/A: not applicable; there were no pregnant participants in the high-severity group.

^c^BMI categories were as follows (kg/m^2^): <18.5 (0), 18.5-22.9 (1), 23.0-24.9 (2), 25.0-29.9 (3), ≥30 (4).

^d^Systolic blood pressure categories were as follows (mm Hg): <120 (0), 120-129 (1), 130-139 (2), 140-159 (3), ≥160 (4).

^e^Diastolic blood pressure categories were as follows (mm Hg): <80 (0), 80-89 (1), 90-99 (2), ≥100 (3).

### Imputation and Standardization

In the data set, some features were missing (Table S1 in Multimedia Appendix 1). To handle the missing data, we calculated the mean value from the training data set for each feature and replaced the missing data with the mean value in both the training and testing data sets. We then performed standardization of the data set, which is a common requirement for machine learning algorithms. The standardization changes the data distribution of each feature with a mean of zero and standard deviation of one:


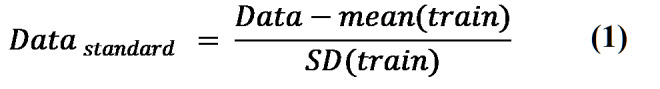


where *mean*(*train*) and *SD*(*train*) are the mean and standard deviation values, respectively, for each feature from the training data set. We applied the standardization to both the training and testing data sets.

### Data Split

For the feature importance analysis and the AI prediction model development, we performed a grid search with a 5-fold cross-validation and 10-time repetition. For that, we divided the 5601 records into training (4480/5601, 80.0%) and testing (1121/5601, 20.0%) data sets in a stratified fashion (Table 3). We used 4480 records as the training data set (4260/4480, 95.1% low severity and 220/4480, 4.9% high severity) and 1121 records as the testing data set (1070/1121, 95.5% low severity and 51/1121, 4.5% high severity). The testing data set was isolated and used only for evaluating the performance of the proposed model.

The training data set (n=4480) was randomly shuffled and partitioned into 5 equal folds in a stratified manner: each fold included 433 low-severity records and 15 high-severity records. Of the 5 folds, a single fold was retained as the validation data set for testing the model, and the remaining 4 folds were used as the training data. We repeated the process 10 times, with each of the 10 folds used exactly once as the validation data. Here, since the number of low-severity records was much higher than the number of high-severity records, we up-sampled the high-severity data by randomly copying the data to prevent the model’s bias toward the low-severity data by balancing the amount of data in the two groups.

**Table 3:** Summary of training and testing data sets.

Data set	Records, n (%)	
	Low-severity group	High-severity group
Training (n=4480)	4260 (95.1)	220 (4.9)
Testing (n=1121)	1070 (95.5)	51 (4.5)
Total (N=5601)	5330 (95.2)	271 (4.8)

### Feature Selection

In order to select important features that influence clinical severity, we first investigated the contribution of each of the 37 input variables on severity via feature importance analysis using AdaBoost [19,20], random forest [21], and eXtreme Gradient Boosting (XGBoost) [22] algorithms. After analyzing the feature importance values from each classifier algorithm, we normalized and averaged the values to calculate the combination feature importance values.

By repeating the 5-fold cross-validation 10 times, we found the best hyperparameters. For AdaBoost, we set the hyperparameters as follows: the number of tree estimators was set to 50 and the learning rate was set to 0.4. For random forest, we set the number of tree estimators to 100, the maximum depth to 4, and the maximum features to 5. For XGBoost, we set the maximum depth to 2, the learning rate to 0.2, the number of tree estimators to 100, the value of the regularization parameter α to 1.0, the fraction of observations to 0.9, and the fraction of columns to 0.9.

The 10-time repeated 5-fold cross-validation provided 50 sets of feature importance values for each classifier (ie, AdaBoost, random forest, and XGBoost). We then averaged the 50 sets of importance values and normalized them so that the importance values from each classifier were in the range from 0 to 1. Finally, we averaged the importance values for the final ranked feature importance value. Moreover, we determined the optimal number of top features to incorporate into the AI prediction model based on the cross-validation results.

### AI Prediction Model Development

To develop the final AI model for severity prediction, we used a deep neural network (DNN). In the DNN approach, we investigated up to 5 hidden layers and each layer depth (ie, node) up to the previous layer depth (ie, node). For the input layer, we first ranked the features according to their importance and increased the number of top features used in the input layer from 1 to 37. For the fully connected (FC) layers as hidden layers, we applied dropouts by changing the dropout rate from 0 to 0.5 with 0.1 increments. The last FC layer was fed into a sigmoid layer, which is an output layer providing the probabilities for the patient severity. We trained the models with the Adam optimizer and binary cross-entropy cost function with a learning rate of 0.0001 and batch size of 64. We implemented the models using R, version 4.0.2 (The R Foundation), with TensorFlow, version 1.13.1, for DNN; scikit-learn, version 0.22.1, for machine learning algorithms; and xgboost, version 0.6.4, for the XGBoost algorithm.

For each set of top features, we found the best cross-validation accuracy using the metrics of area under the curve (AUC) and balanced accuracy:


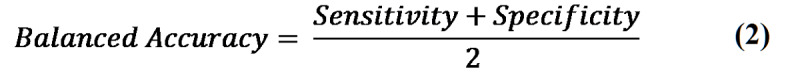


Given the cross-validation accuracy analysis, we finally modeled with the 5-layer DNN using the top 20 features. The 5-layer DNN comprised an input layer, 3 FC layers as hidden layers, and an output layer. The input layer was fed into a series of 3 FC layers consisting of 20, 16, and 8 nodes, respectively. In the first 2 FC layers, we used a dropout rate of 0.5. Then, the last FC layer was fed into a sigmoid layer.

### Performance Evaluation

We evaluated the prediction performance of our proposed 5-layer DNN model with the isolated testing data set (n=1121). To compare the prediction performance of the DNN model with those of other external AI models, we separately trained the following models: logistic regression, decision tree, random forest, support vector machine, XGBoost, AdaBoost, GradBoost, and HistBoost. We evaluated the prediction performance of these AI models as single models as well as ensemble models.

## Results

### Feature Selection

**Figure 1:**
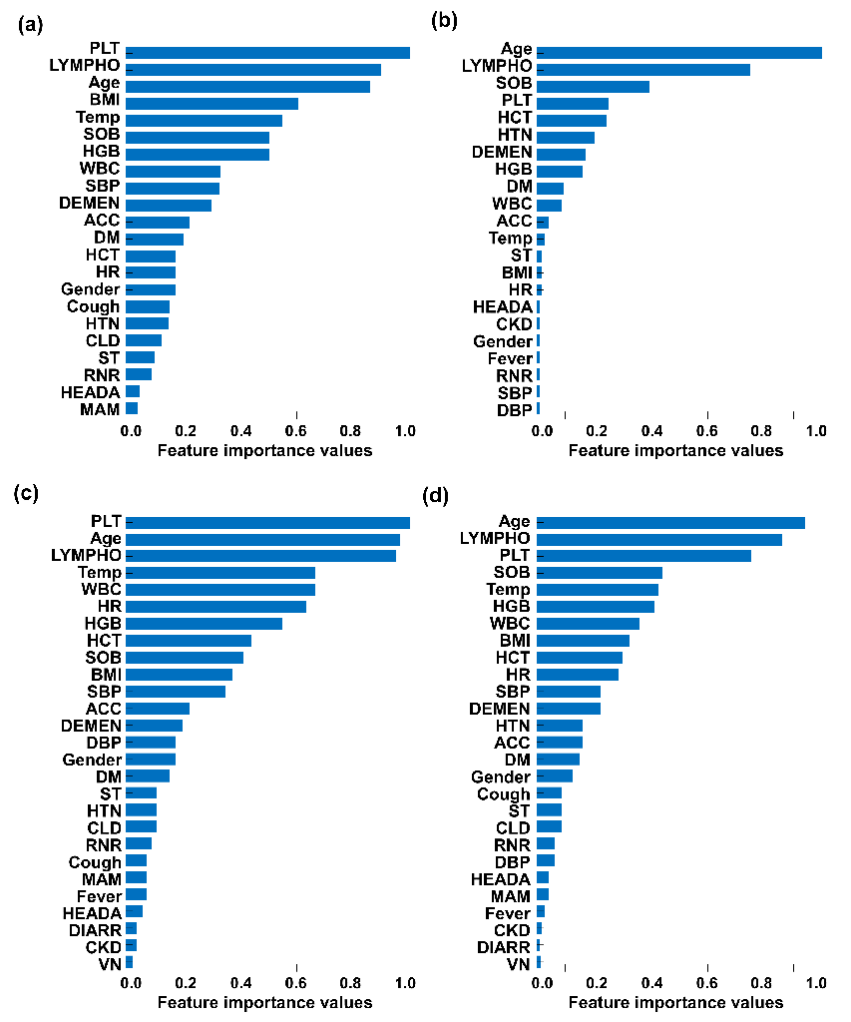
Results of normalized feature importance analysis from (a) AdaBoost, (b) random forest, and (c) eXtreme Gradient Boosting (XGBoost) as well as (d) the combined average ranked feature importance. ACC: altered consciousness/confusion; BMI: body mass index; CCD: chronic cardiac disease; CKD: chronic kidney disease; CLD: chronic liver disease; COPD: chronic obstructive pulmonary disease; DBP: diastolic blood pressure; DEMEN: dementia; DIARR: diarrhea; DM: diabetes mellitus; FM: fatigue/malaise; HCT: hematocrit; HEADA: headache; HF: heart failure; HGB: hemoglobin; HR: heart rate; HTN: hypertension; LYMPHO: lymphocyte; MAM: muscle aches/myalgia; PLT: platelets; Preg: pregnancy; PregWk: pregnancy weeks; RDAD: rheumatism/autoimmune disease; RNR: runny nose/rhinorrhea; SBP: systolic blood pressure; SOB: shortness of breath/dyspnea; SPUTUM: sputum production; ST: sore throat; Temp: temperature; VN: vomiting/nausea; WBC: white blood cells.

Figure 1 shows the results of the ranked feature importance analysis from AdaBoost, random forest, XGBoost, and their combination. Results from AdaBoost indicate that platelet count had the highest importance value, followed by lymphocyte level, age, and body mass index (Figure 1, a). Results from random forest indicate that age had the highest importance value, followed by lymphocyte level, shortness of breath or dyspnea, and platelet count (Figure 1, b). Results from XGBoost indicate that platelet count had the highest importance value, followed by age, lymphocyte level, and temperature (Figure 1, c). By averaging the values obtained from the three models, age had the highest importance value, followed by lymphocyte level, platelet count, and shortness of breath or dyspnea (Figure 1, d). On the other hand, cancer, fatigue or malaise, chronic obstructive pulmonary disease, sputum production, chronic cardiac disease, heart failure, asthma, rheumatism or autoimmune diseases, pregnancy, and pregnancy week rarely contributed to the predictive model. The normalized feature importance values from AdaBoost, random forest, and XGBoost, as well as the combined ranked feature importance values with those averages, are summarized in Table S2 in Multimedia Appendix 1.

**Figure 2:**
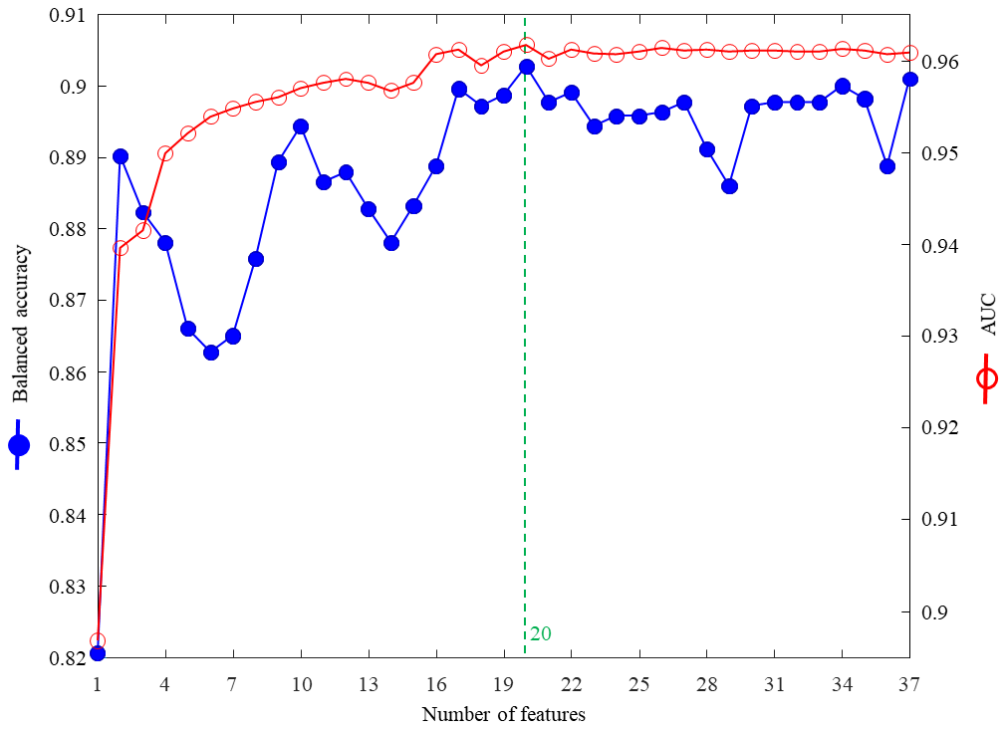
The influence of feature importance values on cross-validation accuracy. AUC: area under the curve.

We investigated the cross-validation performance with the metrics of AUC and balanced accuracy (Figure 2). The results show that both AUC and balanced accuracy reached the highest values when the top 20 features from the combination of AdaBoost, random forest, and XGBoost were used for the input layer. Therefore, we incorporated the top 20 features into the AI prediction model, which yielded a sensitivity of 88%, specificity of 90%, accuracy of 90%, balanced accuracy of 89%, and AUC of 0.96 (Table 4).

**Table 4:** Cross-validation results.

Model	Cross-validation measures (n=448), mean (SD)				
	Sensitivity	Specificity	Accuracy	Balanced accuracy	Area under the curve
5-layer deep neural network	0.88 (0.06)	0.90 (0.02)	0.90 (0.02)	0.89 (0.04)	0.96 (0.01)

### Performance of the AI Prediction Model

With the isolated testing data set (n=1121), our proposed 5-layer DNN showed a sensitivity of 90.20%, specificity of 90.37%, accuracy of 90.37%, balanced accuracy of 90.28%, and AUC of 0.96. Table 5 shows the prediction performances on the testing data set. First, we compared the accuracy metrics when the synthetic minority oversampling technique was applied, and we found that both balanced accuracy and AUC were slightly lower. Second, we compared the accuracy metrics when principal component analysis (PCA)–based feature reduction was applied with eight dimensions, and we found that both balanced accuracy and AUC were also slightly lower. Table 5 [19-28] also shows the prediction performances of various AI models; it can be seen that our proposed 5-layer DNN method provided higher accuracy, balanced accuracy, and AUC values than the other external AI models (ie, logistic regression, decision tree, random forest, support vector machine, XGBoost, AdaBoost, GradBoost, and HistBoost).

Furthermore, we investigated the prediction performance of ensemble AI models (ie, combination of AI models); none of the ensemble AI models outperformed our proposed 5-layer DNN model (Table 6).

**Table 5:** Testing data results and comparison with other machine learning algorithms.

Model	TN^a^	FP^b^	FN^c^	TP^d^	Sen^e^	Spe^f^	Acc^g^	BA^h^	AUC^i^
5-layer DNN^j^: copying	967	103	5	46	0.9020	0.9037	0.9037	0.9028	0.9617
5-layer DNN: SMOTE^k^ [23]	984	86	8	43	0.8431	0.9196	0.9161	0.8814	0.9555
5-layer DNN with PCA^l^ (8 features)	922	148	5	46	0.9020	0.8617	0.8635	0.8818	0.9549
Linear regression [24]	983	87	7	44	0.8627	0.9187	0.9161	0.8907	0.9563
Decision tree [25]	915	155	5	46	0.9020	0.8551	0.8573	0.8786	0.9252
Random forest [21]	955	115	5	46	0.9020	0.8925	0.8930	0.8972	0.9590
Support vector machine [26]	955	115	5	46	0.9020	0.8925	0.8930	0.8972	0.9588
XGBoost^m^ [22]	945	125	6	45	0.8824	0.8832	0.8831	0.8828	0.9558
AdaBoost [19,20]	937	133	5	46	0.9020	0.8757	0.8769	0.8888	0.9586
GradBoost [27]	936	134	6	45	0.8824	0.8748	0.8751	0.8786	0.9525
HistBoost [28]	959	111	7	44	0.8627	0.8963	0.8947	0.8795	0.9535

^a^TN: true negative.

^b^FP: false positive.

^c^FN: false negative.

^d^TP: true positive.

^e^Sen: sensitivity.

^f^Spe: specificity.

^g^Acc: accuracy.

^h^BA: balanced accuracy.

^i^AUC: area under the curve.

^j^DNN: deep neural network.

^k^SMOTE: synthetic minority oversampling technique.

^l^PCA: principal component analysis.

^m^XGBoost: eXtreme Gradient Boosting.

**Table 6:** Test result comparison with ensemble approaches.

Model	TN^a^	FP^b^	FN^c^	TP^d^	Sen^e^	Spe^f^	Acc^g^	BA^h^	AUC^i^
5-layer deep neural network (DNN) (proposed)	967	103	5	46	0.9020	0.9037	0.9037	0.9028	0.9617
DNN + linear regression (LR)	976	94	6	45	0.8824	0.9121	0.9108	0.8973	0.9589
DNN + random forest (RF)	967	103	5	46	0.9020	0.9037	0.9037	0.9028	0.9572
DNN + AdaBoost	965	105	5	46	0.9020	0.9019	0.9019	0.9019	0.9607
DNN + eXtreme Gradient Boosting (XGBoost)	963	107	6	45	0.8824	0.9000	0.8992	0.8912	0.9490
DNN + support vector machine (SVM)	962	108	5	46	0.9020	0.8991	0.8992	0.9005	0.9563
RF + AdaBoost	954	116	5	46	0.9020	0.8916	0.8921	0.8968	0.9515
DNN + RF + AdaBoost	967	103	5	46	0.9020	0.9037	0.9037	0.9028	0.9579
DNN + RF + SVM	962	108	5	46	0.9020	0.8991	0.8992	0.9005	0.9556
DNN + RF + LR	963	107	5	46	0.9020	0.9000	0.9001	0.9010	0.9585
DNN + RF + AdaBoost + XGBoost	944	126	5	46	0.9020	0.8822	0.8831	0.8921	0.9571
DNN + RF + AdaBoost + SVM	959	111	5	46	0.9020	0.8963	0.8965	0.8991	0.9562
DNN + RF + AdaBoost + XGBoost + SVM	978	92	6	45	0.8824	0.9140	0.9126	0.8982	0.9572

^a^TN: true negative.

^b^FP: false positive.

^c^FN: false negative.

^d^TP: true positive.

^e^Sen: sensitivity.

^f^Spe: specificity.

^g^Acc: accuracy.

^h^BA: balanced accuracy.

^i^AUC: area under the curve.

## Discussion

### Principal Findings

Our proposed AI model, the 5-layer DNN using the selected top 20 features, was able to predict the severity of COVID-19 patients at the hospital admission stage with excellent prediction performance: 90.2% sensitivity, 90.4% specificity, and 90.4% accuracy. The model has several unique characteristics. First, it was developed based on nationwide confirmed COVID-19 patient data obtained from the KDCA. In South Korea, all confirmed cases must be reported to the KDCA; thus, the KDCA data are very accurate and updated on a daily basis [4]. The Korean government designated more than 100 general hospitals, including 20 tertiary hospitals, as specialized infection control hospitals equipped with isolation and negative pressure rooms. These designated hospitals should report important clinical information about COVID-19 patients to the KDCA, especially for patients who are admitted to hospitals or show severe conditions. When we were allowed to access the KDCA data sets in September 2020, there were data from 5601 patients with comprehensive clinical information that we could use to develop an AI prediction model. This is the largest cohort with a sufficient amount of data to develop reliable and generalizable AI prediction models.

Second, our AI prediction model development started with feature importance analysis of the 37 features in the comprehensive data set. Of these, 20 were selected based on ranked feature importance analysis results in order to develop an accurate AI prediction model. The cross-validation demonstrated that the AI prediction model showed higher accuracy using the selected 20 features compared to using all 37 features. In addition, the selected 20 features (ie, age, lymphocyte level, platelet count, shortness of breath or dyspnea, temperature, hemoglobin level, white blood cell count, body mass index, hematocrit level, heart rate, systolic blood pressure, dementia, hypertension, altered consciousness or confusion, diabetes, gender, cough, sore throat, chronic liver disease, and runny nose or rhinorrhea) can be easily acquired from patient history, basic physical examinations, and routine laboratory tests. Thus, our AI prediction model can be easily incorporated into routine clinical practice. Furthermore, we observed that PCA-based feature selection also provided as good of a performance as did the feature importance analysis. In particular, we expect that many researchers will be able to flexibly diversify the model for predicting the severity of COVID-19 patients, in that a similar level of accuracy could be obtained with only eight features.

In terms of our feature selection process, we combined AdaBoost, random forest, and XGBoost machine learning algorithms to rank the important features. The AdaBoost algorithm is part of the family of boosting algorithms and sequentially growing decision trees as weak learners [19]. It is well known that it rarely overfits in low-noise data sets [20]. The random forest algorithm is based on a bagging approach, which is based on the aggregation of a set of weak learners [21]. XGBoost is a recently introduced algorithm with optimized gradient boosting [22]. In low-dimensional or highly separable data, all of the classifiers generally provide reasonably good performance. However, they may provide different performances depending on various factors, such as feature dimension, data separability, data balancing, and feature correlation. That is the reason we have combined the three algorithm results.

We named our proposed AI prediction model KOVIDnet, indicating the deep learning algorithm for Korean COVID-19 patients. Owing to its high accuracy and generalizability in Korea, we expect that KOVIDnet will be able to provide treatment priority guidance at the time of admission regarding who should be treated intensively. Although most patients with COVID-19 showed mild and self-limiting illness, some patients became severely and critically ill, showing the rapid progression to acute respiratory failure, sepsis, septic shock, multi-organ failure, and eventual death [29-32]. The mortality rate of severe cases is about 20 times higher than that of mild cases [30,33]. This indicates that early identification of patients at risk of mortality is important for the management of COVID-19 patients.

### Limitations and Future Work

In our earlier study, we grouped the patients into eight subgroups. Subgroup 1 patients had no limits to their activity. Subgroup 2 patients had limits to their activity, but did not need oxygen. Subgroup 3 patients needed oxygen with a nasal prong. Subgroup 4 patients needed oxygen with a facial mask. Subgroup 5 patients needed noninvasive ventilation. Subgroup 6 patients needed invasive ventilation. Subgroup 7 patients had multi-organ failure or underwent extracorporeal membrane oxygenation. Subgroup 8 patients died. For the multiclass classification, we also trained the model through the same procedure as mentioned above, but the accuracy when using the testing data was not satisfactory (Table S3 in Multimedia Appendix 1). It may be because there was no distinct difference in features according to each subgroup or because the number of training data values was insufficient. In addition, the data were extremely imbalanced: the imbalance ratio was 405 (Table S4 in Multimedia Appendix 1). After analyzing the results from the eight-subgroup multiclass classification problem, we considered the binary classification problem, where the low-severity group included subgroups 1 to 4 and the high-severity group included subgroups 5 to 8. Not only was the binary classification problem the most realistic in training the predictive model based on our current data, but it was also useful to convey clinically important implications. We believe that we can extend our model to the multiclass classification problem based on more extensive data.

Our study also has additional limitations. First, our proposed AI prediction model was validated with an isolated test data set (n=1121), which was a data set that was split from the entire data set. It may be necessary to validate our AI model with external data sets, such as prospectively collected data. To validate and update KOVIDnet, we made a web application [34] so that anyone can access the model. We believe that sharing the AI model with the public will be helpful in validating and improving its performance. Second, our data did not include patients of other races, such as Caucasian or Middle East Asian. In the near future, we have a plan to apply our AI model to various data sets, including data from patients of other races. To realize this goal, we will establish a real-time training framework that can train our model using prospectively collected data from all over the world. We believe that we can improve KOVIDnet for better generalization based on the extended data.

### Conclusions

In conclusion, we developed our AI model with 20 selected features based on a large nationwide data set, and it was able to predict the severity of COVID-19 accurately. We believe that our model can help health care providers to effectively treat COVID-19 patients at an early stage and ultimately reduce deaths.

## Appendix

Multimedia Appendix 1 Supplementary tables.

## Abbreviations

AI: artificial intelligence
AUC: area under curve
DNN: deep neural network
FC: fully connected
KDCA: Korea Disease Control and Prevention Agency
NRF: National Research Foundation of Korea
PCA: principal component analysis
XGBoost: eXtreme Gradient Boosting
