# Supplementary material for: Prediction and Feature Importance Analysis for Severity of COVID-19 in South Korea Using Artificial Intelligence: Model Development and Validation
Source: J Med Internet Res. 2021 Apr 19;23(4):e27060. doi: 10.2196/27060 (PMC8057199; doi:10.2196/27060)
Supplement: Multimedia Appendix 1 [file jmir_v23i4e27060_app1.docx]

Table S1. Summary of missing data for 37 features from 5,601 data.

| **No** | **Features** | **The number of patients missing the feature (percentage)** | |
| --- | --- | --- | --- |
| **1** | Age | 0 | (0.00%) |
| **2** | Gender | 0 | (0.00%) |
| **3** | Pregnancy | 20 | (0.36%) |
| **4** | Pregnancy week | 5583 | (99.68%) |
| **5** | BMI | 1201 | (21.44%) |
| **6** | SBP | 142 | (2.54%) |
| **7** | DBP | 142 | (2.54%) |
| **8** | HR | 129 | (2.30%) |
| **9** | Temperature | 42 | (0.75%) |
| **10** | Fever | 4 | (0.07%) |
| **11** | Cough | 4 | (0.07%) |
| **12** | SPUTUM | 4 | (0.07%) |
| **13** | ST | 4 | (0.07%) |
| **14** | RNR | 4 | (0.07%) |
| **15** | MAM | 4 | (0.07%) |
| **16** | FM | 4 | (0.07%) |
| **17** | SOB | 4 | (0.07%) |
| **18** | HEADA | 4 | (0.07%) |
| **19** | ACC | 4 | (0.07%) |
| **20** | VN | 4 | (0.07%) |
| 21 | DIARR | 4 | (0.07%) |
| 22 | DM | 3 | (0.05%) |
| 23 | HTN | 3 | (0.05%) |
| 24 | HF | 3 | (0.05%) |
| 25 | CCD | 19 | (0.34%) |
| 26 | Asthma | 3 | (0.05%) |
| 27 | COPD | 3 | (0.05%) |
| 28 | CKD | 3 | (0.05%) |
| 29 | Cancer | 4 | (0.07%) |
| 30 | CLD | 326 | (5.82%) |
| 31 | RDAD | 332 | (5.93%) |
| 32 | DEMEN | 329 | (5.87%) |
| 33 | HGB | 1521 | (27.16%) |
| 34 | HCT | 1526 | (27.25%) |
| 35 | LYMPHO | 1545 | (27.58%) |
| 36 | PLT | 1520 | (27.14%) |
| 37 | WBC | 1520 | (27.14%) |

Table S2. Normalized feature importance from Adaboost, RF and XGBoost, and the ranked feature importance with those average.

| **No** | **Features** | **AdaBoost** | **RF** | **XGBoost** | **Three model** |
| --- | --- | --- | --- | --- | --- |
| **1** | Age | 0.86 | 1.00 | 0.96 | 0.94 |
| **2** | LYMPHO | 0.89 | 0.75 | 0.95 | 0.86 |
| **3** | PLT | 1.00 | 0.25 | 1.00 | 0.75 |
| **4** | SOB | 0.51 | 0.39 | 0.41 | 0.44 |
| **5** | Temperature | 0.55 | 0.03 | 0.67 | 0.42 |
| **6** | HGB | 0.51 | 0.16 | 0.55 | 0.41 |
| **7** | WBC | 0.33 | 0.08 | 0.67 | 0.36 |
| **8** | BMI | 0.61 | 0.02 | 0.37 | 0.33 |
| **9** | HCT | 0.18 | 0.24 | 0.44 | 0.29 |
| **10** | HR | 0.18 | 0.02 | 0.63 | 0.28 |
| **11** | SBP | 0.32 | 0.01 | 0.35 | 0.22 |
| **12** | DEMEN | 0.30 | 0.17 | 0.20 | 0.22 |
| **13** | HTN | 0.15 | 0.21 | 0.11 | 0.16 |
| **14** | ACC | 0.22 | 0.04 | 0.22 | 0.16 |
| **15** | DM | 0.21 | 0.09 | 0.15 | 0.15 |
| **16** | Gender | 0.18 | 0.01 | 0.17 | 0.12 |
| **17** | Cough | 0.16 | 0.00 | 0.07 | 0.08 |
| **18** | ST | 0.10 | 0.02 | 0.11 | 0.08 |
| **19** | CLD | 0.13 | 0.00 | 0.11 | 0.08 |
| **20** | RNR | 0.09 | 0.01 | 0.09 | 0.06 |
| 21 | DBP | 0.01 | 0.01 | 0.17 | 0.06 |
| 22 | HEADA | 0.05 | 0.01 | 0.06 | 0.04 |
| 23 | MAM | 0.04 | 0.00 | 0.07 | 0.04 |
| 24 | Fever | 0.00 | 0.01 | 0.07 | 0.03 |
| 25 | CKD | 0.02 | 0.01 | 0.04 | 0.02 |
| 26 | DIARR | 0.00 | 0.00 | 0.04 | 0.01 |
| 27 | VN | 0.00 | 0.00 | 0.02 | 0.01 |
| 28 | Cancer | 0.00 | 0.00 | 0.01 | 0.00 |
| 29 | FM | 0.00 | 0.00 | 0.01 | 0.00 |
| 30 | COPD | 0.00 | 0.00 | 0.01 | 0.00 |
| 31 | SPUTUM | 0.00 | 0.00 | 0.01 | 0.00 |
| 32 | CCD | 0.00 | 0.00 | 0.01 | 0.00 |
| 33 | HF | 0.00 | 0.00 | 0.00 | 0.00 |
| 34 | Asthma | 0.00 | 0.00 | 0.00 | 0.00 |
| 35 | RDAD | 0.00 | 0.00 | 0.00 | 0.00 |
| 36 | Pregnancy | 0.00 | 0.00 | 0.00 | 0.00 |
| 37 | Pregnancy week | 0.00 | 0.00 | 0.00 | 0.00 |

Table S3. Testing data results from multiclass classification problems with eight subgroups

| Subgroup | TN | FP | FN | TP | Sensitivity | Specificity | Accuracy | Balanced accuracy |
| --- | --- | --- | --- | --- | --- | --- | --- | --- |
| 1 | 162 | 56 | 284 | 619 | 0.6855 | 0.7431 | 0.6967 | 0.7143 |
| 2 | 888 | 171 | 46 | 16 | 0.2581 | 0.8385 | 0.8064 | 0.5483 |
| 3 | 937 | 94 | 59 | 31 | 0.3444 | 0.9088 | 0.8635 | 0.6266 |
| 4 | 1,068 | 44 | 8 | 1 | 0.1111 | 0.9604 | 0.9536 | 0.5358 |
| 5 | 1,103 | 12 | 5 | 1 | 0.1667 | 0.9892 | 0.9848 | 0.5780 |
| 6 | 1,113 | 3 | 5 | 0 | 0.0000 | 0.9973 | 0.9929 | 0.4987 |
| 7 | 1,116 | 1 | 4 | 0 | 0.0000 | 0.9991 | 0.9955 | 0.4996 |
| 8 | 1,033 | 46 | 14 | 28 | 0.6667 | 0.9574 | 0.9465 | 0.8120 |
| mean |  |  |  |  | 0.2791 | 0.9242 | 0.9050 | 0.6016 |

Subgroup 1 patients had no limit of activity. Subgroup 2 patients had limit of activity, but did not need oxygen. Subgroup 3 patients needed oxygen with a nasal prong. Subgroup 4 patients needed oxygen with a facial mask. Subgroup 5 patients needed non-invasive ventilation. Subgroup 6 patients needed invasive ventilation. Subgroup 7 patients had multi-organ failure or underwent extracorporeal membrane oxygenation (ECMO). Subgroup 8 patients died.

Table S4. The number of patients in each subgroup for multiclass classification problem

| Subgroup | 1 | 2 | 3 | 4 | 5 | 6 | 7 | 8 | Total |
| --- | --- | --- | --- | --- | --- | --- | --- | --- | --- |
| Number of patients | 4,455 | 330 | 469 | 43 | 33 | 19 | 11 | 241 | 5,601 |

Subgroup 1 patients had no limit of activity. Subgroup 2 patients had limit of activity, but did not need oxygen. Subgroup 3 patients needed oxygen with a nasal prong. Subgroup 4 patients needed oxygen with a facial mask. Subgroup 5 patients needed non-invasive ventilation. Subgroup 6 patients needed invasive ventilation. Subgroup 7 patients had multi-organ failure or underwent extracorporeal membrane oxygenation (ECMO). Subgroup 8 patients died.
